# Using Ensemble OCT-Derived Features beyond Intensity Features for Enhanced Stargardt Atrophy Prediction with Deep Learning

**DOI:** 10.3390/app13148555

**Published:** 2023-07-24

**Authors:** Zubin Mishra, Ziyuan Wang, SriniVas R. Sadda, Zhihong Hu

**Affiliations:** 1 Doheny Image Analysis Laboratory, Doheny Eye Institute, Pasadena, CA 91103, USA; 2 School of Medicine, Case Western Reserve University, Cleveland, OH 44106, USA; 3 Electrical and Computer Engineering, University of California, Los Angeles, CA 90095, USA; 4 Department of Ophthalmology, David Geffen School of Medicine, University of California, Los Angeles, CA 90095, USA

**Keywords:** biomedical optical imaging, ensemble deep convolutional neural networks, optical coherence tomography, advanced OCT-derived features, data augmentation and enhancement, predictive models

## Abstract

Stargardt disease is the most common form of juvenile-onset macular dystrophy. Spectral-domain optical coherence tomography (SD-OCT) imaging provides an opportunity to directly measure changes to retinal layers due to Stargardt atrophy. Generally, atrophy segmentation and prediction can be conducted using mean intensity feature maps generated from the relevant retinal layers. In this paper, we report an approach using advanced OCT-derived features to augment and enhance data beyond the commonly used mean intensity features for enhanced prediction of Stargardt atrophy with an ensemble deep learning neural network. With all the relevant retinal layers, this neural network architecture achieves a median Dice coefficient of 0.830 for six-month predictions and 0.828 for twelve-month predictions, showing a significant improvement over a neural network using only mean intensity, which achieved Dice coefficients of 0.744 and 0.762 for six-month and twelve-month predictions, respectively. When using feature maps generated from different layers of the retina, significant differences in performance were observed. This study shows promising results for using multiple OCT-derived features beyond intensity for assessing the prognosis of Stargardt disease and quantifying the rate of progression.

## 1. Introduction

Stargardt disease is a recessive inherited disorder that is the most common form of juvenile-onset macular dystrophy, causing progressive damage or degeneration of the macula [1–8]. Fundus autofluorescence (FAF) imaging and spectral-domain optical coherence tomography (SD-OCT) are two widely accessible imaging modalities that can aid in the diagnosis, monitoring, and classification of Stargardt disease. FAF imaging provides an in vivo assay of the lipofuscin content within the retinal pigment epithelium (RPE) cells. However, this does not provide any direct measure of the anatomical status of the photoreceptors [9]. On the other hand, SD-OCT allows for the three-dimensional visualization of the retina’s microstructure and the direct evaluation of individual retinal layers, including the photoreceptors and RPE [10,11].

In SD-OCT, Stargardt disease manifests as disruption of the outer retinal layers, namely the photoreceptor inner and outer segments and RPE. At a Retinal Disease Endpoints meeting with the Food and Drug Administration (FDA) in November of 2016, the integrity of the ellipsoid zone (EZ) was proposed as a reliable measure of the anatomic status of the photoreceptors and a suitable regulatory endpoint for therapeutic intervention clinical trials [12].

Stargardt disease is currently phenotypically classified by appearance on various imaging modalities, including FAF and SD-OCT [13–16]. The rate of progression does not specifically factor into these classifications. Similarly, approaches for automated and semiautomated analysis of features associated with Stargardt disease focus on analysis at a single point in time [17–21]. Approaches to the automated analysis of Stargardt disease frequently make use of some variants of U-Nets. U-Nets are a state-of-the-art deep convolutional neural network (CNN) architecture for semantic segmentation [22]. CNN-based models have achieved state-of-the-art performance in several eye-assessment tasks [23–27]. However, they are difficult to interpret, and it is often unclear how their final decisions are reached, making CNNs something of a black-box representation [28]. An extension of CNNs to address this issue are ensemble CNNs, where multiple neural networks are used together to each handle different inputs. This approach has been used in the segmentation and prediction of geographic atrophy in age-related macular degeneration [29,30].

This study, as a preliminary investigation, seeks to use baseline OCT en face feature maps generated from SD-OCT segmentation for the predictive modeling of future atrophy in Stargardt disease using deep convolutional neural networks. Typically, in the field of disease detection and analysis, OCT en face intensity maps are used [30–32]. In our approach, we apply multiple advanced en face feature maps beyond intensity maps to augment and enhance data with the goal of enhancing the algorithm’s performance. This paper represents the first attempt to use such enhanced en face feature maps generated from disease-relevant retinal layers segmented from OCT to predict future Stargardt atrophy. Furthermore, we report an inherently interpretable ensemble neural network architecture designed to allow the determination of what features or combinations of features contribute to the development and progression of atrophy in Stargardt disease.

## 2. Materials and Methods

### 2.1. Imaging Dataset

A total of 264 eyes from 155 patients diagnosed with Stargardt disease were identified from the ProgStar study that had both SD-OCT scans performed on the initial visit and FAF imaging performed at a six-month follow-up. Of these two hundred and sixty-four eyes, two hundred and thirty-seven were identified that had FAF imaging performed at a twelve-month follow-up. All of the FAF images were manually segmented by certified graders to create ground-truth masks marking the atrophied regions of the retina. Images were randomly assigned to training and testing sets in an approximate 3:1 ratio. Of the six-month follow-up FAF images, two hundred of the images were used for training, and sixty-four were used for testing. Of the twelve-month follow-up FAF images, one hundred and eighty of the images were used for training, and fifty-seven of the images were used for testing. The SD-OCT volume dimensions are 496 (depth) × 1024 (A-scans) × 49 (B-scans) pixels or 496 (depth) × 512 (A-scans) × 49 (B-scans) pixels. The 496 × 512 images were resized to a standard width of 1024. After segmentation, 49 × 1024 feature maps were produced from each SD-OCT volume. These feature maps were resized to 512 × 512 for registration to FAF images and later resized to 128 × 128 prior to being input to the neural network due to memory constraints. SD-OCT scans and FAF images of right (OD) eyes were reflected horizontally to maintain consistency throughout training and testing.

All images were de-identified according to the Health and Insurance Portability and Accountability Act Safe Harbor. All subjects gave their informed consent far inclusion before they participated in this study. This study was conducted in accordance with the Declaration of Helsinki, and the protocol was approved by the Ethics Committee of each of the participating institutions. This study has been registered at https://www.clinicaltrials.gov (NCT01977846, accessed on 12 June 2023).

### 2.2. Image Registration

**Figure 1.**
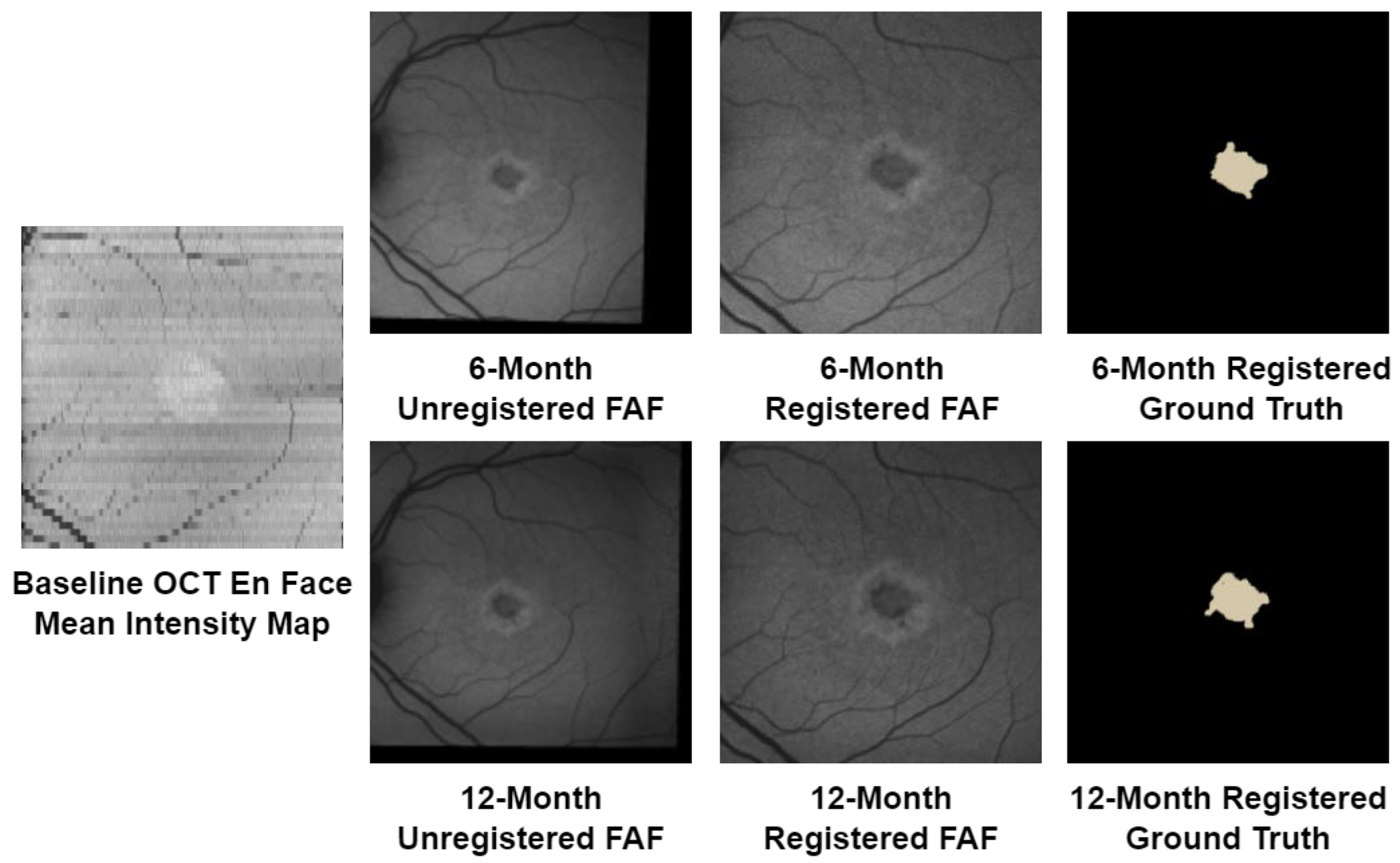
FAF and manual ground-truth registration. The baseline OCT en face mean intensity map shows vessel features that were used to register the shown six-month and twelve-month FAF images and ground truths.

Registration of FAF images and ground-truth manual segmentations was completed using OCT en face mean intensity feature maps of the layers between the external limiting membrane (ELM) and tire Bruch’s membrane, which provided visualization of vessels that could be used as landmarks for the registration. The OCT en Pace map was generated from the baseline SD-OCT scans, resulting in feature maps of 49 × 1024, which were then rasized to 512 × 512 for ease of viewing. FAF images and ground-truth segmentation marking the region of atrophy from the six-month and twelve-month follow-up datasets were then registered to this baseline feature map through feature-based image registration, using vessel branching, bifurcation, and crossover points to determine corresponding points in the images. Figure 1 shows an example of a baseline OCT en face map) and the registrations of follow-up FAF images and ground truths to it.

### 2.3. Neural Network Structure

**Figure 2.**
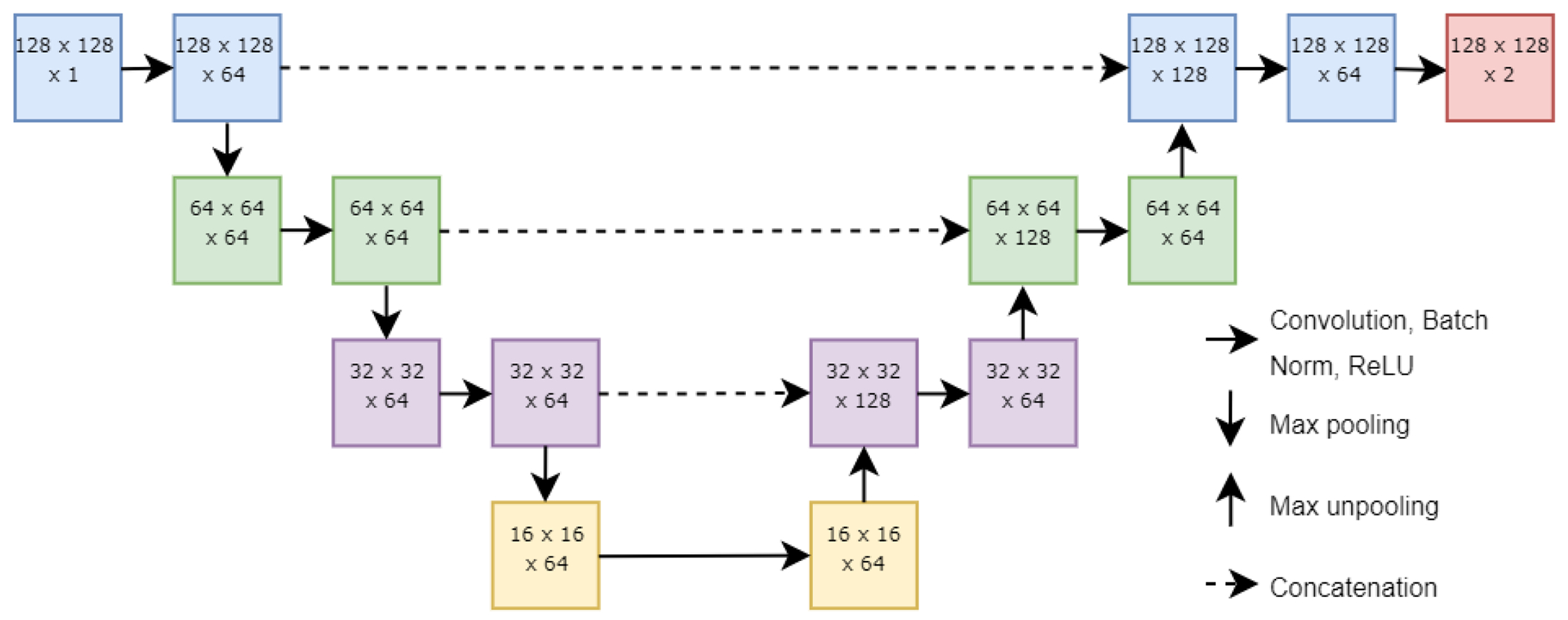
Architecture of a single U-Net in the ensemble neural network. Each solid horizontal arrow represents a set of a convolutional, a batch normalization, and a ReLU layer; each solid down arrow represents a max pooling layer; each up arrow represents a max unpooling layer; and each dashed horizontal arrow represents concatenation. The numbers in each box represent the dimensions of the data in that layer (height × width × number of channels).

The ensemble neural network used in this study is made of a modular series of U-Nets, a state-of-the-art deep) learning architecture for semantic stgmentation that can be trained using very few images [22,23]. Each component U-Net consists of a series of encoder blocks followed by a series of decoder blocks. Each encoder block conaists of a convolutional, a batch) normalization, a rectified linear unit (ReLU), and a max pooling layet. Each decoder block consists of a max unpooling, a concatenation, a convolutional, a batch normalization, and a ReLU layer. Concatenation is performed with feature maps from the corresponding encoder block. All convolution kernels were set at a size of 5 × 5, with a 1 × 1 kernel to complete the binary classification task. Of note, there is no softmax layer in the component U-Nets. Rather, the maximum activations are obtained from each component U-Net after the binary classification, and this result is sent to a softmax layer to calculate the probabilities for each pixel to be assigned to the two classes of atrophy or not atrophy. The architecture of a single U-Net is shown in Figure 2.

**Figure 3.**
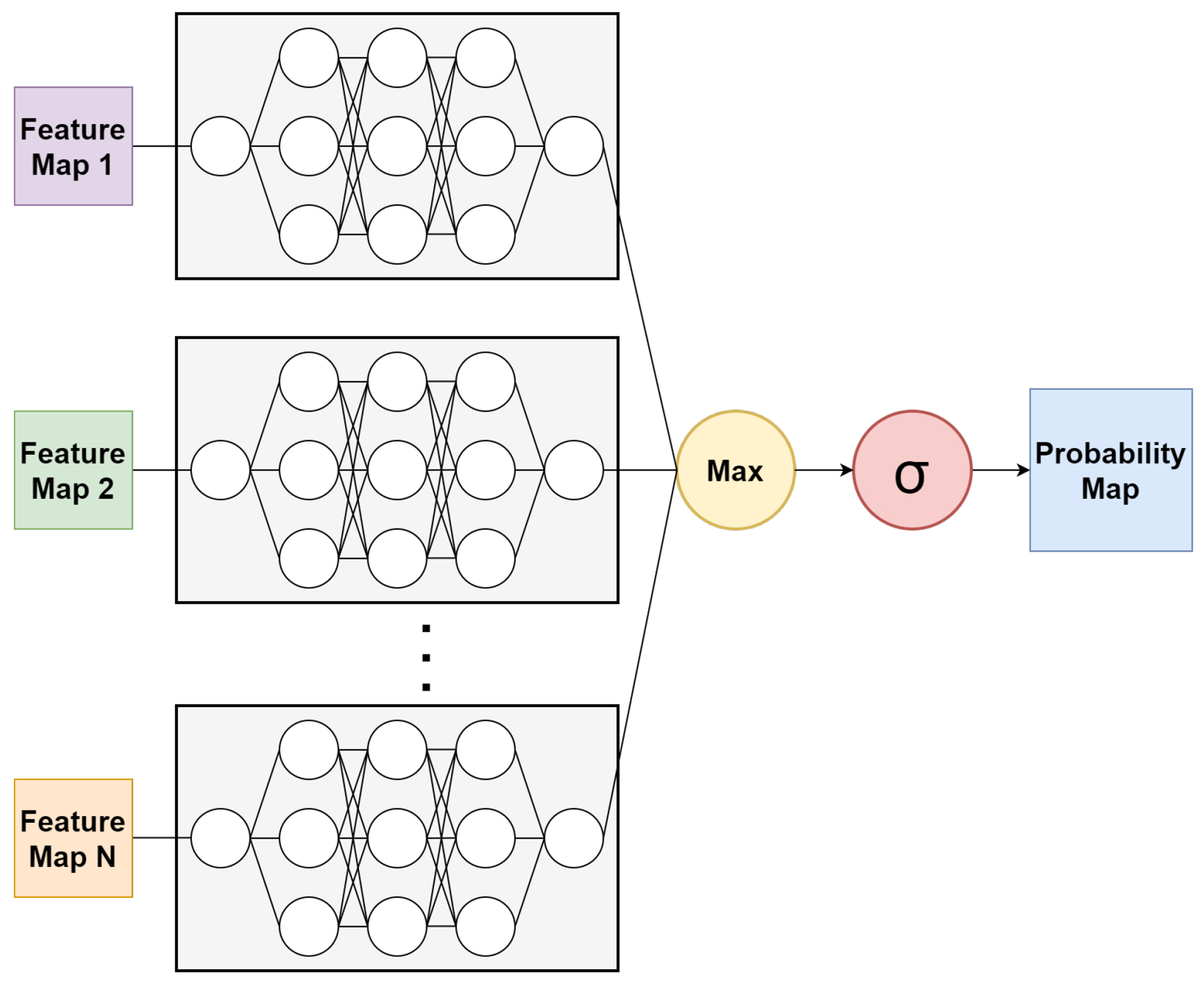
Architecture of the ensemble neural network. Each component U-Net takes in a single feature map as input. The outputs of all the U-Nets are combined through a maximum function, and probability maps are produced using a softmax function.

Figure 3 shows the architecture of the ensemble neural network used in the prediction of atrophy from baseline feature maps. Each U-Net takes in one feature map as input. Such a strategy naturally augments data, and the various advanced features derived from OCT potentially enhance data beyond the commonly used mean intensity features. After the 1 × 1 convolution for binary classification, the outputs of the U-Nets are combined by taking the maximum activations for each pixel from the component U-Nets. This can be imagined as follows:

(1) $$g\left(E\left(Y\right)\right)=\max\left(f_{1}\left(x_{1}\right),f_{2}\left(x_{2}\right),\ldots ,f_{n}\left(x_{n}\right)\right)$$

where $x_{1},x_{2},\ldots ,x_{n}$ is the input with n features, Y is the observed quantity to be approximated, g(⋅) is the link function, and each f(⋅) stands for a component U-Net. These maximum activations are subsequently sent to a softmax function to obtain probability maps of the area of interest. These probability maps can be used as-is or binarized through thresholding. The U-Nets are trained jointly through back-propagation.

The neural network is optimized using weighted two-class logistic loss, Dice loss, and a weight decay term for regularization:

(2) $${ℒ}_{Logloss}=-\sum_{x\in \text{Ω}}w\left(x\right)g_{l}\left(x\right)\log\left(p_{l}\left(x\right)\right)$$

(3) $${ℒ}_{Dice}=1-\frac{2\sum_{x\in \text{Ω}}p_{l}\left(x\right)g_{l}\left(x\right)}{\sum_{x\in \text{Ω}}{g_{l}}^{2}\left(x\right)+\sum_{x\in \text{Ω}}{p_{l}}^{2}\left(x\right)}$$

(4) $${ℒ}_{Overall}={ℒ}_{Logloss}+{ℒ}_{Dice}+\lambda {\left\|W\right\|}_{F}^{2}$$

In the above equations, w(x) is the weight assigned to pixel x∈Ω, $g_{l}\left(x\right)$ is the ground-truth probability that pixel x belongs to class l, $p_{l}\left(x\right)$ is the estimated probability that pixel x belongs to class l, λ is the weight decay, and ${\left\|W\right\|}_{F}$ is the Frobenius norm on the weights of the neural network. The neural network has a total parameter count of 8.3 million and 89.7 GFLOPs.

This ensemble neural network structure allows for the contributions of each feature map to be isolated and interpreted. Furthermore, this structure makes it a simple matter to add or remove features from consideration by adding or removing the component modular U-Nets.

**Figure 4.**
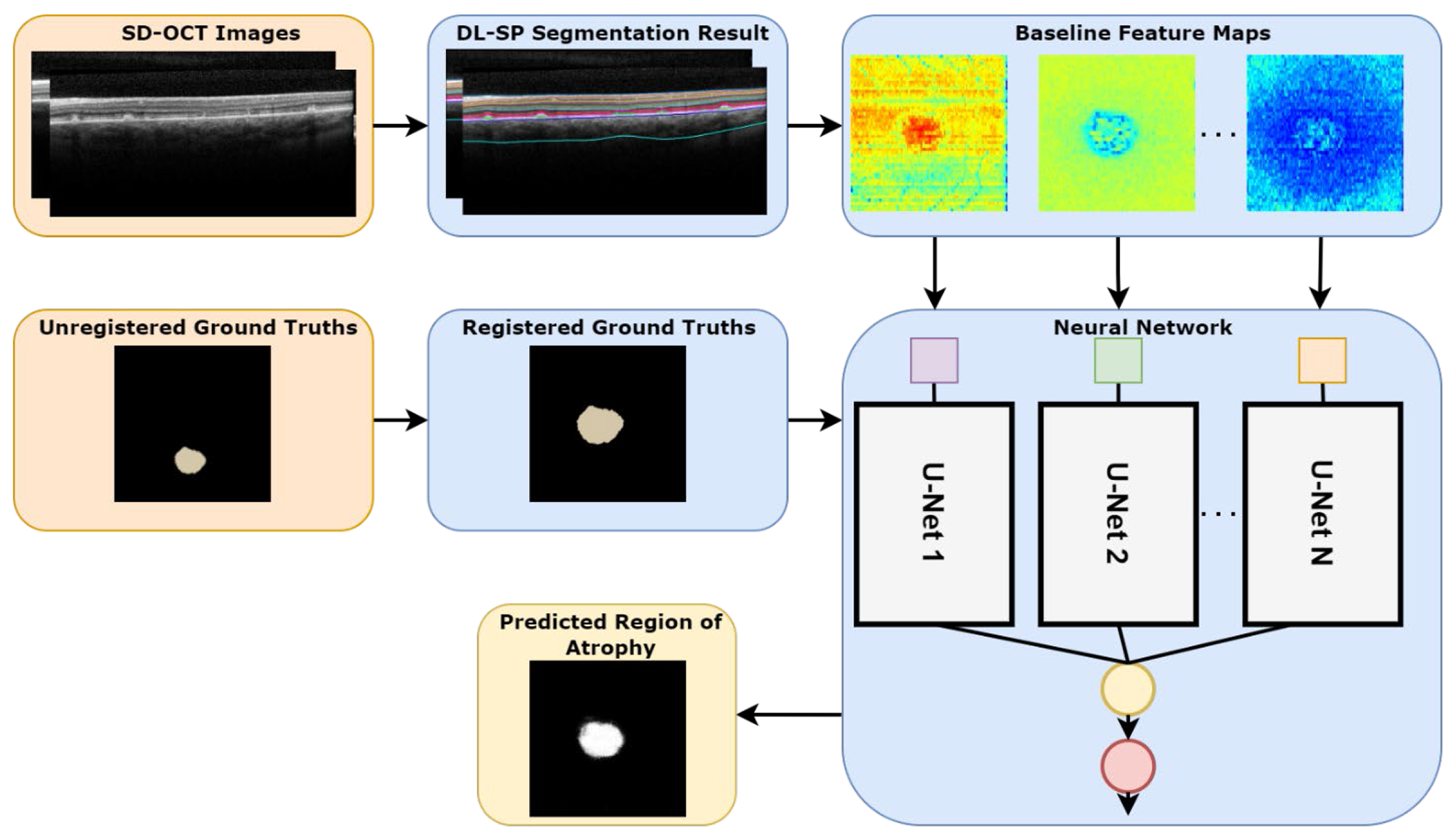
Overview of the approach to predicting the region of atrophy in Stargardt patients. Orange boxes represent inputs, blue boxes represent processing and training, and yellow boxes represent output.

Figure 4 is an overview of the entire Stargardt progression prediction system, starting with baseline SD-OCT segmentation and registration of six-month and twelve-month FAF images and ground truths. The baseline SD-OCT segmentation results in a number of feature maps that serve as inputs to an ensemble neural network, with each component U-Net taking one feature map as input. The neural network was trained using the registered six-month and twelve-month manual segmentations as ground truths. The output of the ensemble neural network is a probability map for the predicted region of atrophy.

The training was conducted using stochastic mini-batch gradient descent with a batch size of 8. The learning rate was set to 10^−2^ initially for 40 epochs and 10^−3^ for the final 20 epochs for 60 total epochs. The momentum was set at 0.95 with a weight decay of 10^−4^. Pixel weights were assigned as 6 for the atrophied region and background and 15 for the boundary between them. Training and testing took approximately 45 s and 4 s per epoch, respectively. The neural network was implemented using MATLAB R2017a and was run on a desktop PC with an Intel i7-7800X CPU, 16 GB NVIDIA Quadro P5000 GPU, and 32 GB RAM.

### 2.4. Experimental Methods

The initial visit SD-OCT scans for each image were segmented using the Deep Learning–Shortest Path (DL-SP) algorithm previously developed by our group [17,33]. From the results of this segmentation, feature maps were generated using various segmented layers as boundaries. These feature maps included mean intensity, median intensity, maximum intensity, minimum intensity, standard deviation, skewness, kurtosis, and thickness. The SD-OCT scan dimensions resulted in feature maps of 49 × 1024, which were resized to 512 × 512 for the purposes of registration before being downscaled to 128 × 128 to meet the memory constraints faced when training the neural network.

After registration of the FAF images, the previously mentioned feature maps were generated for the regions between the ELM and ellipsoid zone (EZ), the EZ and inner retinal pigment epithelium, the inner RPE and outer RPE, the outer RPE and choroid–sclera (C–S) junction, the EZ and inner RPE, and ± 5 pixels from the EZ. For the regions between the outer RPE and the C-S junction and ± 5 pixels from the EZ, the thickness feature map was excluded. This was because of too much natural variation in the case of the outer RPE to C-S junction thickness and because there would be a constant thickness in the case of ±5 pixels from the EZ.

The registered FAF images and ground truths and the generated initial visit feature maps were then sent to the neural network described above. The neural network output a probability of the predicted region of atrophy based on the initial visit SD-OCT scan feature maps. The accuracy of the probability map was graded using the Dice coefficient [34]:

(5) $$D=\frac{2\sum_{x\in \text{Ω}}p_{l}\left(x\right)g_{l}\left(x\right)}{\sum_{x\in \text{Ω}}{g_{l}}^{2}\left(x\right)+\sum_{x\in \text{Ω}}{p_{l}}^{2}\left(x\right)}$$

This measures the degree of overlap between the prediction and the ground truth. The activations of each component U-Net were also extracted. These activations were sent through a sigmoid function and were also graded using the Dice coefficient to determine which feature maps were the best and worst contributors to the final result. The pixel accuracy of the predictions was also calculated.

In addition, neural networks using only the traditional mean intensity feature maps were also created for the purpose of comparison.

## 3. Results

The accuracy of the predicted regions of atrophy was evaluated using the median Dice coefficient and pixel accuracy of the eyes tested. Tables 1 and 2 show the median Dice coefficients and median pixel accuracies for the prediction of atrophy at a six-month follow-up for feature maps generated from several different regions. Tables 3 and 4 show the same for a twelve-month follow-up. Also included in these tables are the Dice coefficients and pixel accuracies calculated from the activations of the individual component U-Nets after being passed through a sigmoid function.

From these results, it was examined whether using multiple feature maps resulted in significant improvement and whether the choice of region had a significant impact on performance. For each of the ELM-EZ, EZ-IRPE, IRPE-ORPE, ELM-IRPE, and EZ ± 5 regions, the Wilcoxon signed-rank test resulted in *p*-values less than 0.05 for both six-month and twelve-month predictions when comparing both Dice coefficient and pixel accuracy for neural networks using all feature maps versus neural networks using only the mean intensity feature map, indicating a significant difference in performance when adding additional feature maps. For a comparison between the choice of regions, the ELM-IRPE and EZ ± 5 regions were examined as representative of two different strategies for selecting a region. Significant differences were found when comparing Dice coefficients for both six-month and twelve-month predictions when using all feature maps but not when using only one feature map. These findings were not reflected when comparing pixel accuracy, with significant differences found for six-month predictions when using all features or only mean intensity and for twelve-month predictions when using only mean intensity, while no significance was found when all features were used. These results for six-month and twelve-month predictions are shown in Tables 5 and 6.

**Figure 5.**
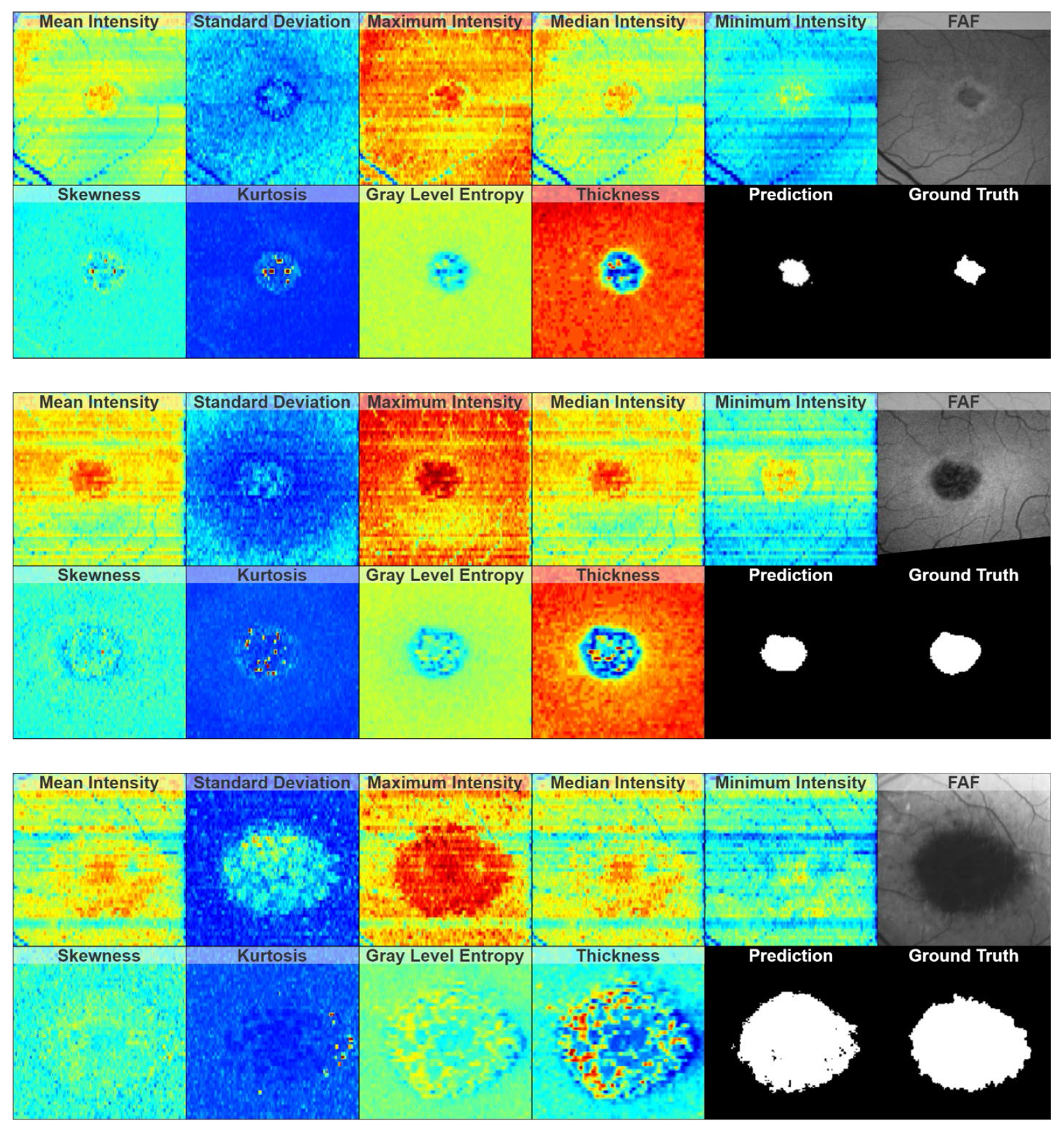
Examples of ELM-IRPE feature maps, resultant six-month atrophy prediction, and comparison to the corresponding six-month FAF and ground truth for a relatively mild (**top**), a relatively moderate (**middle**), and a relatively severe (**bottom**) case of atrophy.

**Figure 6.**
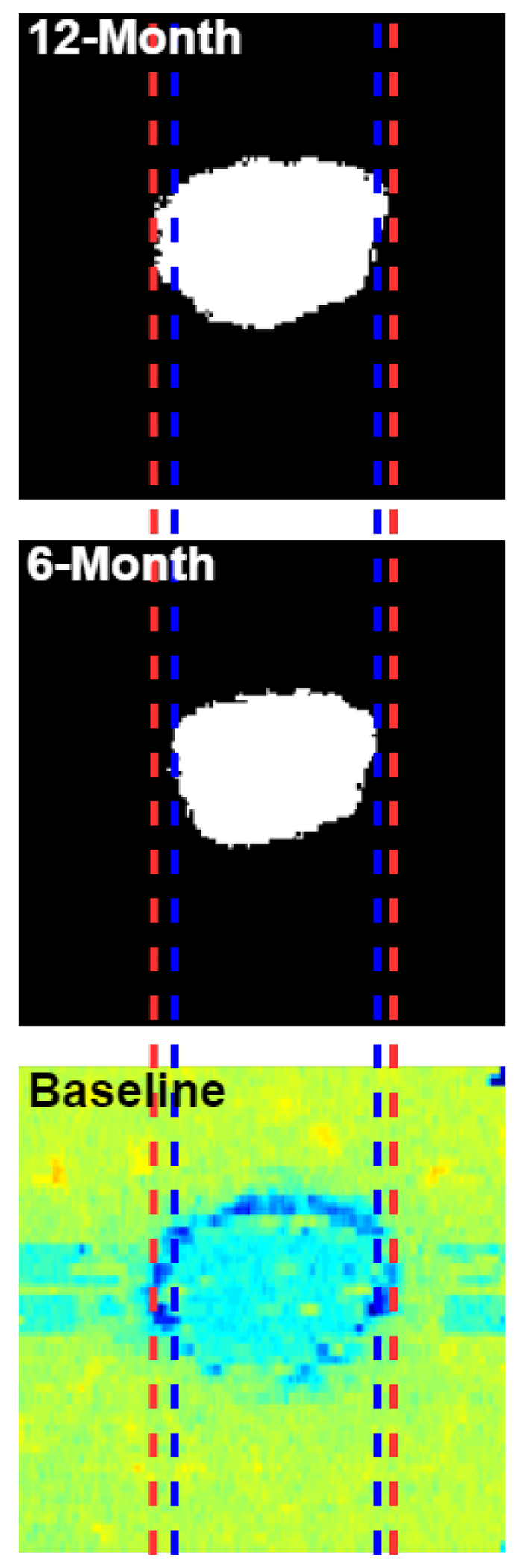
A comparison of six-month and twelve-month predictions to a baseline feature map (gray-level entropy). The blue and red lines indicate the breadth of the six-month and twelve-month predictions, respectively. They indicate an increase in predicted area and where that increase in area corresponds on the baseline feature maps.

**Figure 7.**
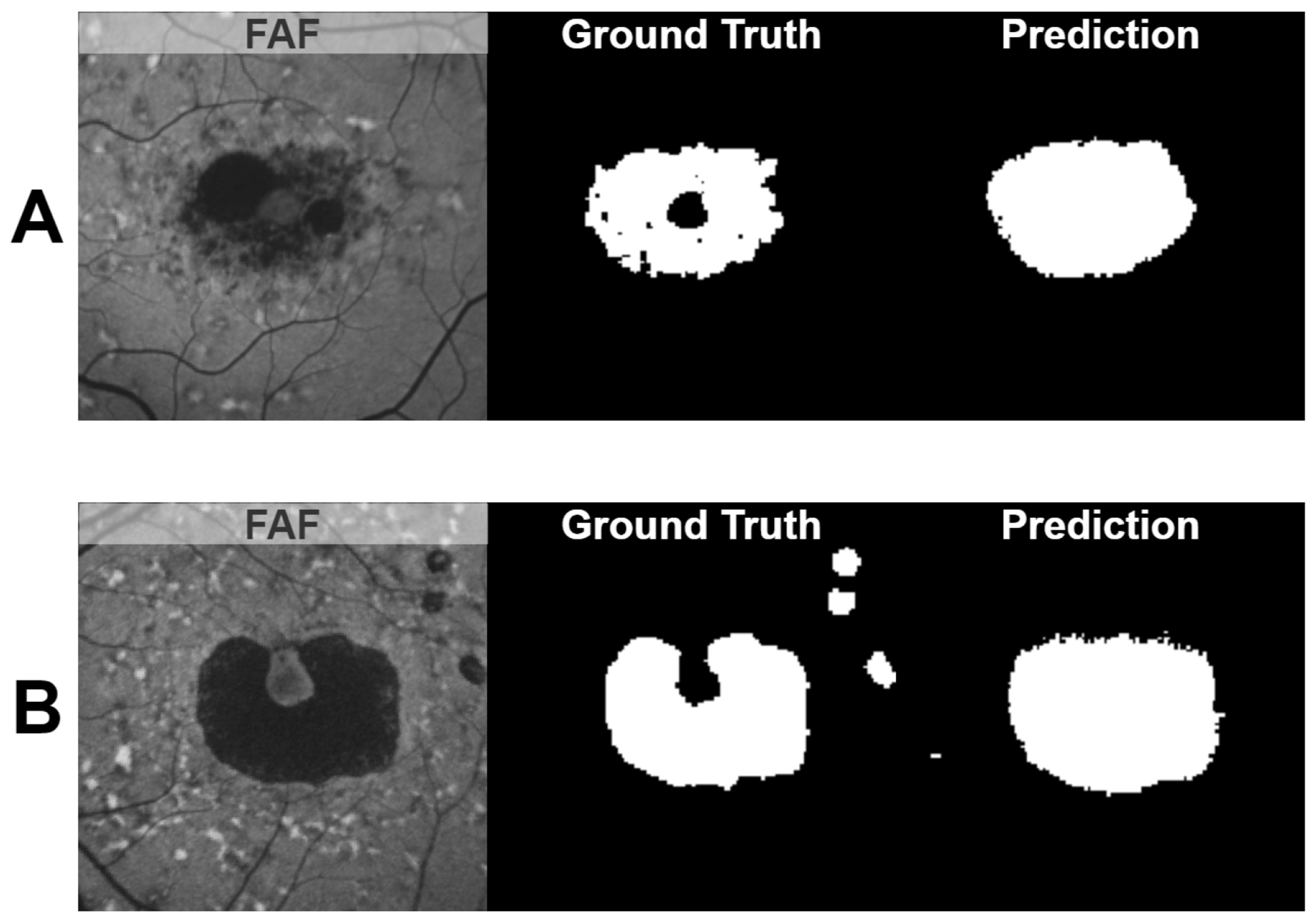
Examples of two of the commonly encountered segmentation errors in this study. In both (**A**) and (**B**), the predicted atrophy fills the hole in the ground truth. In (**B**), the predicted atrophy does not include the satellite areas of atrophy in the ground truth.

Examples of predicted regions of atrophy and their corresponding ELM-IRPE feature maps are shown in Figure 5. These examples show a comparison of input feature maps, output prediction maps, and ground truth. This figure also showcases the range of sizes of atrophied regions included in the dataset. In Figure 6, the six-month and twelve-month predictions are shown for the same eye, alongside a comparison to one of the baseline feature maps. In Figure 7, two of the common errors in segmentations that were noted are shown: filling holes in the prediction map compared to the ground truth and not segmenting satellite lesions.

## 4. Discussion and Conclusions

This paper represents the first attempt to uce novel en face feature maps generated from disease-relevant retinal layers segmented in baseline SD-OCT images to predict future Stargardt atrophy.

Previous approaches for automated and semiautomated analysis of features associated with Statgardt disease generally focus on analysis at a single point in time [17–21]. For example, J. Kugelman developed an approach using a U-Net-inspired neural network and graph search algorithm to segment the retinal boundaries in SD-OCT images. J. Charng developed an approach uiing a U-Net model with ResNet encoding steps to identify hyperautofluorescent flecks on FAF imagee. In an extension of J. Kugelman’s approach, we devuloped an approach using U-Net models and graph search algorithms to segment multiple retinal layers and Stargardt features in SD-OCT images. In other previous work, our group used ResNet to develop an automated screening system for Stargardt atrophy and U-Net to segment the atrophic lesions from FAF images. P. Zhao developed an approach to multilabel segmentation of FAF images of Stargardt atrophy, performing automated segmentation of multiple autofluorescence lesion types using a modified U-Net using ResNet encoder blocks. None of these approaches have considered an attempt to predict this prouression of Stargardt disease. The one other attempt to predict the progression of Stargardt disease using deep) learning methohs also comes from our groups, in which a self-attended U-Net was developed to predict file progression of atrophy twelve months from a baseline FAF image [35]. The self-attention mechanism used allowed for the identification of early signs of disease progression towards atrophy, capturing “feature rings” corresponding with atrophy progression. With all of the previously mentioned methods, FAF images were used. However, FAF images can be thought of as a 2D summary of a 3D structure. Not only. is there a loss of data in that transformation, but also, there can tie inclusion of data from irrelevant parts of the 3D structure. With SD-OCT images, it is possible to look at the entire 3D structure and selectively decide what to then include in the 2D representation. In the field of the research of a different macular atrophic disease, i.e., geographic atrophy prediction in late-stage age-related macular degeneration, a related problem, Anegondi et al. developed a model using FAF and SD-OCT en face images to predict annualized atrophy growth rate using the Inception v3 architecture [36]. In their approach, OCT en face images were generated using mean intensity over full, sub-Bruch’s membrane, and above-Bruch’s membrane areas. However, they did not look at any more specific regions of the retina (e.g., the ellipsoid zone), and the only feature they used to generate en face maps was mean intensity. A final approach to atrophy prediction, again seen in the context of geographic atrophy, is described by Zhang et al. [37]. In their approach, longitudinal data are able to be leveraged using bidirectional long short-term memory (LSTM) based prediction module. Their approach made use of the full volumetric OCT data at multiple visits to predict future atrophy, additionally making use of a 3D U-Net to refine the final segmentation. Their approach avoids the potential loss of axial information that comes from the generation of 2D en face maps.

The ensemble neural network presented in this paper achieved a median Dice coefficient of 0.830 for six-month predictions of atrophy against the ground truth and 0.828 for twelve-month predictions of atrophy against the ground truth. Significant improvement was observed when comparing neural networks that used only the mean intensity feature map and the ensembled neural networks that used several additional feature maps (*p* < 0.05). Significant differences in performance were also observed when using the ELM-IRPE region to generate feature maps compared to using the EZ ± 5 region (*p* < 0.05). These differences were observed for both the six-month and twelve-month predictions.

The Dice scores achieved are also an improvement from our previous work [35], which reported a 12-month prediction Dice score of 0.76. Notably, our previous work used FAF images, while this work uses en face maps generated from SD-OCT images. The use of SD-OCT images allows us to focus on disease-relevant retinal layers in a way that is not possible with FAF images. This may explain the improved performance.

In Tables 1–4, the highest performances are seen in the ELM-EZ, EZ-IRPE, IRPE-ORPE, ELM-IRPE, and EZ ± 5 regions, with weaker performance in the ORPE-C-S regions. This falls in line with the pattern of Stargardt disease in SD-OCT imaging, namely that disruption is frequently seen at the EZ junction and the RPE. Within these five regions, Dice coefficients remain around 0.8 for both the six-month and twelve-month predictions, indicating that, from the baseline images, predictions can be made for at least up to twelve months later, possibly longer.

The Dice coefficients and pixel accuracies calculated from the activations of the individual component U-Nets shown in Tables 1–4 can be thought of as a measure of that feature’s strength or contribution. In almost all cases, mean intensity performs as a relatively weak feature, with other, less commonly used features frequently standing out as stronger predictors, such as skewness, kurtosis, and standard deviation. Interestingly, the strength of features can differ depending on which region of the retina was used to generate the feature maps and between six-month and twelve-month predictions. For example, when looking at Dice coefficients, for six-month predictions, the ELM-EZ region has kurtosis as the strongest feature, and the ELM-IRPE region has gray-level entropy as the strongest feature, while for twelve-month predictions, the ELM-EZ region’s strongest feature changes to skewness but the ELM-IRPE region’s strongest feature remains gray-level entropy. This indicates that the optimal feature maps for prediction may differ depending on the retinal region being examined and how far into the future the prediction is being made. Also of note is that when looking at pixel accuracy, different features appear to contribute more strongly than when looking at Dice coefficients; however, because the neural network was trained in part using Dice loss, we believe the Dice coefficients are a better representation of each features relative strength.

In Figure 5, it is possible to compare the final prediction of the ensemble neural network to the input feature maps generated from baseline and the FAF and ground truth from follow-up. Of interest in these images is that while minimum intensity appears to be the feature map in which the area of atrophy is least distinguishable, it is not the lowest-performing feature map, as shown in Tables 1–4. This indicates that feature maps may not be able to be treated independently when deciding which feature maps are most contributory. The inclusion of a group of weak-appearing feature maps may outperform a single feature map that appears strong or more clearly defined.

In Figure 6, an increase in the predicted area of atrophy can be seen from the six-month to the twelve-month prediction, as indicated by the orange lines. They appear to correspond to the inner and outer borders of a ring that can be seen on the baseline gray-level entropy feature map. This provides evidence that this neural network is capable of predicting up to twelve months into the future and that predictions for different time points are indeed different.

In Tables 5 and 6, significant differences can be seen when comparing performance between using one feature and using several features. Furthermore, when using several features, a significant difference is seen in the performances between regions used to generate feature maps. However, notably, when only one feature is used, there is no significant difference between the regions used. This indicates that using additional features accentuates the difference between different regions during analysis.

In Figure 7, examples of two of the commonly encountered segmentation errors in this study are shown. In both Figure 7A,B, the predicted atrophy fills the hole that is present in the ground truth. In Figure 7B, the predicted atrophy does not include the satellite areas of atrophy in the ground truth. We suspect that this is because, in general, the atrophic regions in the dataset used are contiguous, central, and elliptical without holes. As such, the neural network has not been trained on enough data to consistently recognize when there is a hole in the atrophic region or when there are regions of atrophy outside of the center of the image.

This study is not without limitations. Although this is a larger dataset than most cohorts of Stargardt disease, the sample size is still limited. Due to the relative scarcity of data on Stargardt disease, it is difficult to capture all aspects of the disease within a deep learning model, and human graders struggle with making determinations in the process of grading. This is exemplified in the difficulties faced when segmenting such phenotypes as poorly demarcated questionably decreased autofluorescence [38]. Other limitations include segmentation errors and errors introduced through the registration process, where the registered image may be slightly sheared, rotated, or translated compared to the OCT enface image.

Additionally, in comparing the neural network described to other current state-of-the-art neural networks used in retinal segmentation tasks, we found that the computational complexity of our model is relatively high with regard to floating-point operations (FLOPs). This is shown in Table 7. The increased complexity comes from the modular nature of the neural network. The complexity scales linearly from the computational complexity of a single U-Net with the number of features used.

Future projects would ideally address these limitations and expand the analysis further into how to select the best feature maps to use. While this study shows that the inclusion of several feature maps results in better performance than using only one, each additional feature map increases the computational workload significantly. Therefore, it would be ideal to have a method of selecting the feature maps that contribute the most to the accurate prediction of atrophy. We believe that the design of the ensemble neural network and its inherent interpretability with its separation of input feature maps to individual U-Nets will aid in this task. Other future work would include examining predictions at eighteen-month and later follow-ups and determining at what point, reliable prediction is no longer possible from the baseline images. Additionally, the performance of other architectures could be explored, such as variational auto-encoders to enhance data augmentation, long short-term memory networks to incorporate longitudinal data, and EfficientNet to reduce computational complexity. Furthermore, other imaging modalities, such as FAF and multi- or hyperspectral images could be examined. Multi- and hyperspectral imaging in particular captures more spectral information than imaging modalities like FAF and SD-OCT, and as such may provide further opportunities to identify unique features of disease processes [44,45]. Lastly, such an approach could also potentially be adapted to the detection of other retinal diseases in OCT with enhanced performance.

This study shows promising results for the development of tools for the predicting of the progression of Stargardt disease, providing insight into the potential use of multiple feature maps beyond the traditionally used mean intensity feature maps to improve performance. While this study is a preliminary investigation, it offers the possibility of differentiating patients with Stargardt disease based on predicted progression rate which may be a new approach to phenotypic differentiation. As treatments for Stargardt disease are developed, such a classification may be useful to clinicians in selecting patients for treatment.

## Figures and Tables

**Table 1.** Ensemble neural network and component U-Net median Dice coefficients for six-month prediction.

	ELM-EZ	EZ-IRPE	IRPE-ORPE	ORPE-C-S	ELM-IRPE	EZ ± 5
Ensemble	0.824	0.804	0.803	0.706	0.830	0.803

Mean Intensity	0.196	0.126	0.344	0.259	0.349	0.260
Standard Deviation	0.558	0.694	0.199	0.443	0.303	0.430
Maximum Intensity	0.236	0.241	0.324	0.405	0.161	0.076
Minimum Intensity	0.425	0.089	0.297	0.179	0.327	0.077
Median Intensity	0.341	0.283	0.252	0.267	0.098	0.113
Kurtosis	0.771	0.240	0.592	0.293	0.451	0.219
Skewness	0.479	0.521	0.257	0.410	0.445	0.508
Gray-Level Entropy	0.247	0.276	0.413	0.016	0.677	0.237
Thickness	0.677	0.360	0.687		0.568	

**Table 2.** Ensemble neural network and component U-Net median pixel accuracy for six-month prediction.

	ELM-EZ	EZ-IRPE	IRPE-ORPE	ORPE-C-S	ELM-IRPE	EZ ± 5
Ensemble	0.977	0.973	0.974	0.964	0.980	0.971

Mean Intensity	0.921	0.834	0.873	0.902	0.845	0.854
Standard Deviation	0.902	0.958	0.885	0.897	0.782	0.881
Maximum Intensity	0.940	0.741	0.943	0.923	0.793	0.848
Minimum Intensity	0.920	0.677	0.894	0.889	0.912	0.792
Median Intensity	0.901	0.590	0.928	0.835	0.649	0.709
Kurtosis	0.971	0.842	0.933	0.887	0.858	0.823
Skewness	0.961	0.939	0.809	0.792	0.914	0.892
Gray-Level Entropy	0.947	0.895	0.853	0.808	0.948	0.686
Thickness	0.950	0.856	0.940		0.910	

**Table 3.** Ensemble neural network and component U-Net median Dice coefficients for twelve-month prediction.

	ELM-EZ	EZ-IRPE	IRPE-ORPE	ORPE-C-S	ELM-IRPE	EZ ± 5
Ensemble	0.809	0.828	0.801	0.696	0.823	0.791

Mean Intensity	0.327	0.213	0.312	0.458	0.421	0.180
Standard Deviation	0.684	0.606	0.294	0.377	0.498	0.411
Maximum Intensity	0.244	0.373	0.296	0.276	0.380	0.180
Minimum Intensity	0.427	0.060	0.152	0.328	0.301	0.074
Median Intensity	0.309	0.172	0.191	0.327	0.123	0.068
Kurtosis	0.640	0.376	0.528	0.321	0.405	0.172
Skewness	0.751	0.467	0.245	0.369	0.403	0.544
Gray-Level Entropy	0.624	0.530	0.405	0.082	0.606	0.280
Thickness	0.552	0.421	0.638		0.585	

**Table 4.** Ensemble neural network and component U-Net median pixel accuracy for twelve-month prediction.

	ELM-EZ	EZ-IRPE	IRPE-ORPE	ORPE-C-S	ELM-IRPE	EZ ± 5
Ensemble	0.970	0.973	0.973	0.964	0.977	0.970

Mean Intensity	0.860	0.818	0.929	0.925	0.848	0.911
Standard Deviation	0.927	0.927	0.798	0.836	0.898	0.867
Maximum Intensity	0.898	0.927	0.946	0.924	0.900	0.845
Minimum Intensity	0.852	0.920	0.929	0.878	0.837	0.892
Median Intensity	0.855	0.884	0.942	0.873	0.649	0.839
Kurtosis	0.964	0.811	0.911	0.872	0.801	0.663
Skewness	0.962	0.948	0.932	0.809	0.906	0.905
Gray-Level Entropy	0.898	0.885	0.824	0.830	0.920	0.723
Thickness	0.942	0.880	0.918		0.902	

**Table 5.** Mean intensity vs. many features for six-month prediction.

	ELM-IRPE	EZ ± 5	*p*-Value
All Features Dice Coefficient	0.830	0.803	0.005
Mean Intensity Dice Coefficient	0.744	0.734	0.193
*p*-value	<0.001	<0.001	

All Features Pixel Accuracy	0.980	0.971	0.039
Mean Intensity Pixel Accuracy	0.967	0.968	0.046
*p*-value	<0.001	0.011	

**Table 6.** Mean intensity vs. many features for twelve-month prediction.

	ELM-IRPE	EZ ± 5	*p*-Value
All Features Dice Coefficient	0.823	0.791	0.003
Mean Intensity Dice Coefficient	0.762	0.700	0.395
*p*-value	<0.001	<0.001	

All Features Pixel Accuracy	0.977	0.970	0.076
Mean Intensity Pixel Accuracy	0.969	0.956	0.003
*p*-value	0.001	0.001	

**Table 7.** Computational complexity of deep learning models.

	Total Parameters (M)	FLOPs (G)
U-Net [22]	118.4	35.3
U-Net++ [39]	138.0	87.2
AttentionUNet [40]	54.6	67.2
IPN [41]	103.9	54.1
IPN V2 [42]	207.8	195.6
ARA-Net (EfficientNet) [43]	57.7	7.2

Modular U-Net	8.3	89.7

## Data Availability

The datasets generated and/or analyzed during the current study are not publicly available due to the patients’ privacy and the violation of informed consent but are available from the corresponding author upon reasonable request.

## Featured Application:

This study shows promising results for the development of artificial intelligence tools for the predicting of the progression of Stargardt disease. It further offers the possibility of differentiating patients with Stargardt disease based on predicted progression rate which may be a new approach to phenotypic differentiation or classification that may be useful in clinical decision-making.
